# Sexual Behaviours and Practices before and after Phase I/II HIV Vaccine Trial: A Qualitative Study among Volunteers in Dar es Salaam Tanzania

**DOI:** 10.3390/ijerph17197193

**Published:** 2020-10-01

**Authors:** Masunga K. Iseselo, Edith A. M. Tarimo, Eric Sandstrom, Asli Kulane

**Affiliations:** 1 Department of Clinical Nursing, Muhimbili University of Health and Allied Sciences, Dar es Salaam P.O. Box 65001, Tanzania; 2 Department of Nursing Management, Muhimbili University of Health and Allied Sciences, Dar es Salaam P.O. Box 65001, Tanzania; etarimo54@yahoo.co.uk; 3 Equity and Health Policy Research Group, Department of Global Public Health, Karolinska Institutet, Tomtebodavägen 18A, 171 77 Stockholm, Sweden; asli.kulane@ki.se; 4 Department of Clinical Science and Education, Södersjukhuset, Karolinska Institutet, Tomtebodavägen 18A, 171 77 Stockholm, Sweden; eric.g.sandstrom@gmail.com

**Keywords:** sexual behavior, phase I/II HIV vaccine trial, Tanzania

## Abstract

There is limited information about sexual behavior among volunteers who participated in phase I/II human immunodeficiency virus (HIV) vaccine trial. This article describes the sexual behavior, practices before, and after participation in phase I/II HIV vaccine trial in Dar es Salaam, Tanzania. We conducted a qualitative descriptive study involving volunteers who participated in the phase I/II vaccine trial between 2007 and 2010. Purposeful sampling was used to recruit potential informants. Twenty-four in-depth interviews were conducted. The audio-recorded interviews were transcribed verbatim and analyzed using a thematic content analysis approach. The findings revealed that before participation in the HIV vaccine trial, informants were engaging in unprotected multiple sexual relationships. After the completion of the HIV vaccine trial, informants reported strengthened marital relationships, increased understanding of safer sexual practices, and HIV testing. However, the informants reported challenges regarding vaccine-induced seropositivity that adversely affected their sexual and marital relationships. Some informants re-engaged in risky sexual practices because they perceived the experimental vaccine was protective. The informants suggested having continued interventions within the community to enhance safer sexual practices. Participation in phase I/II HIV vaccine trials may positively and negatively influence changes in volunteers’ sexual behaviors and practices. The trial interventions appear to improve compliance with safer sexual practices. However, the reported vaccine-induced seropositivity and the perception that experimental vaccines are protective need further appropriate interventions.

## 1. Introduction

Human immunodeficiency virus (HIV) continues to be a major public health concern. In 2018, an estimated 37.9 million people were living with HIV, with a global HIV prevalence of 0.8% among adults. The majority of people living with HIV are from low- and middle- income countries, with an estimated 68% living in sub-Saharan Africa [1]. About 1.6 million people were living with HIV in Tanzania in 2018. This equates to an estimated HIV prevalence among adults of 4.8% [2]. In the same year, 72,000 people were newly infected with HIV, and 24,000 people died from an Acquired Immunodeficiency Syndromes (AIDS)-related illness. New HIV infection is contributed by risky sexual behavior among the at-risk population. The consistent use of condoms continues to be advocated for primary prevention of HIV infection despite limited evidence regarding its effectiveness in blocking the sexual transmission of HIV since the past three decades [3]. Lack of knowledge on HIV prevention methods and the high level of misconception pose a need to intensify the education programs on safer sex and HIV/AIDS prevention methods [4]. Behavioral intervention programs focusing on risky sexual behavior reduction need to be strengthened.

The behavioral interventions aim to reduce behaviors that make individuals more vulnerable to HIV infection. These interventions have generally focused to increase the use of condoms or reduce the number of sexual partners [5]. Behavioral interventions such as abstinence and being faithful to one sexual partner [6,7] and condom use [3,8] have been promoted for a long time and remain important factors for HIV prevention in many parts of the world. However, inadequate evidence to support the effectiveness of the behavioral interventions have been reported in Sub-Saharan countries [6,9]. Thus, treatment as prevention (TasP) and the vaccine is more likely to lead to infection control than a single approach [10]. TasP involves prescribing antiretroviral (ARV) HIV drugs to those who are living with HIV to reduce the amount of virus in their blood to undetectable levels so that there is effectively no risk of transmission of HIV [11]. However, the development of an effective preventive HIV vaccine is the most likely promising method of preventing new HIV infections.

Studies on the HIV vaccine trials have shown a significant reduction in risky sexual behaviors among participants [12,13]. This is an important goal in HIV prevention research. Nevertheless, studies exploring the effects of HIV vaccine trials on HIV transmission risk sexual behavior are inconclusive. There are mixed results on the effects of participation in the HIV vaccine trial on participants’ sexual practices [14,15,16]. Some studies report that participants are likely to increase their HIV transmission risk behavior if they receive a hypothetical HIV vaccine [17,18] and others have reported no increase in risky sexual behavior after vaccination [19,20]. A household survey of adults in Uganda reported that a high percentage of respondents would not use a condom for extra-marital sexual partners if they received a hypothetical HIV vaccine [21]. This raises the concerns to HIV vaccine researchers that the ultimate goals of HIV vaccine development may be hindered or delayed if this challenge is not well addressed among the vaccine trial participants.

In addition, participants in preventive HIV vaccine trials may experience negative social consequences of trial participation, including problems related to vaccine-induced seropositivity (VISP) [22,23] and some may persist as long as 23 years [24]. Anecdotal evidence reveals disruption in personal and marital relationships after testing positive as a result of their participation in the HIV vaccine trial [25]. However, these challenges remain unaddressed despite the personal and social harms that cause among the participants of HIV vaccine trials [26]. VISP must be well assessed and evaluated to see how the sexual relationship of trial participants is affected particularly when his/her HIV status is required to be disclosed.

A wish to engage in unprotected sex without fear of infection has been mentioned as a motivator to participate in HIV vaccine trials among high-risk men and women [27]. People may engage in risky sexual behavior after recruitment in the studies with the perception that the experimental vaccine will protect them against HIV infection. Effective training before, during, and after the trial is required for the participants to reduce risky sexual behavior. For example, a phase I/II HIV vaccine trial (HIVIS 03) in Dar es Salaam, Tanzania reported a reduction in risky sexual behavior in most of the participants after frequent training sessions and prolonged counseling and support during the trial [28]. However, some volunteers engaged in unprotected sexual practices despite the frequent education and counseling sessions provided during the trial period. These findings raise the need for a more in-depth assessment of safer sexual practices among HIVIS 03 volunteers who completed phase I/II HIV vaccine trials in Dar es Salaam. Thus, an in-depth exploration of sexual behavior among volunteers is crucial to ensure safer sexual practices are maintained after the HIV vaccine trial.

This study assessed the sexual behavior and practice of volunteers before and 10 years after participation in phase I/II HIV vaccine trial in Dar es Salaam, Tanzania.

## 2. Materials and Methods

### 2.1. Design, Setting, and Population

This was a qualitative descriptive study design. The study was conducted in Dar es Salaam, Tanzania in 2019. We recruited informants who participated in HIVIS 03, a phase I/II HIV vaccine trial between 2007 and 2010. All the informants were police officers [29,30]. Police officers were chosen for this particular HIV vaccine trial because they were considered a low-risk population and most of them had secondary education capable of making independent informed decisions [31].

### 2.2. Sampling and Sample Size

Purposeful sampling was used to recruit potential informants. This method was used based on selecting information-rich cases to yield insights and an in-depth understanding of the participants’ sexual behavior and practices experience. Sampling was conducted through the research assistant who provided the list of participants who fully participated in the HIVIS 03 vaccine trial project. The first author reviewed the list of those who met the inclusion criteria and developed a schedule for an interview with those who fulfilled the criteria. The research assistant invited the potential informants who were available for the interview using their phone numbers in the database. We selected potential informants who were available in Dar es Salaam during the study period. The sample size was determined by the principles of saturation. That is, we terminated sampling when no new information was elicited by sampling more units as proposed by Malterud et al. [32]. Thus, we reached saturation after recruiting 24 participants.

### 2.3. Data Collection 

We collected data through in-depth interviews at the HIV vaccine trial site at Muhimbili National Hospital (MNH), Dar es Salaam. We used a semi-structured interview guide developed in Kiswahili, translated into English, and back-translated into Kiswahili. Kiswahili is the national language spoken by most Tanzanians. The study aimed to explore the sexual behavior and practices among volunteers before and after HIV vaccine trials. The interview guide consisted of the following questions:

1. Why do you think people of your age engage in sexual relationships?
2. What is your current experience in a sexual relationship?
3. What is your experience in the use of protection against HIV and other sexually transmitted infections (STIs)?
4. What is your perception concerning HIV or other STIs testing?
5. To what extent was the information about safer sex practice beneficial to you during and after HIV vaccine trials?
6. What challenges did you face to maintain safer sexual practices after participation in HIV vaccine trials?

We developed these questions based on the previous findings [29,30] to get a deeper understanding of the participants’ sexual behavior before and after the trial. Specific probes followed these questions. We frequently reviewed the interview guide to reflect the emerging themes during the interview. The first author interviewed the participants in a well-lit room that allowed observation of the non-verbal behavior of informants. In addition, the room had minimal noises from the external environment. This allowed maximum exploitation of the information and provided comfortability to the informant. All informants agreed to be audio recorded to catch up with all the information provided. The interviews lasted between 25 to 50 min.

### 2.4. Data Analysis

**Figure 1:**
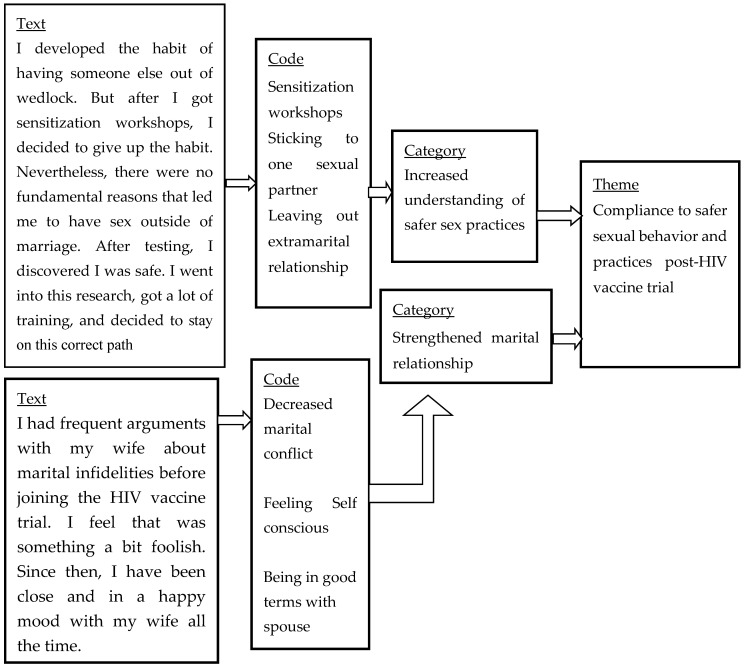
Example of the coding process and formation of a theme.

An experienced research assistant [nurse] transcribed the audio-recorded data verbatim. The first author listened to the audio recorder and counterchecked by reading each transcript to ensure completeness. He corrected discrepancies such as missing words/phrases, and typographical errors. The first two authors extensively read the raw data in the transcripts to get a deeper understanding of the dataset. The content analysis approach as proposed by Graneheim [33] was used to analyze the data. The unit of analysis was the whole textual data obtained from the in-depth interviews. We used inductive coding approaches to analyze the data in the original language (Kiswahili) to preserve the informants’ contextual meaning. To enhance the credibility of the analysis, the first and second authors independently coded the transcripts. The first author used Nvivo 12 (a computer software) to facilitate the coding process, while the second author used manual coding on the margins of the transcripts. Both authors developed categories from meaning units or actual phrases used in specific text segments. Overlapping segments of the texts were coded in more than one category. Within each category, we searched for subtopics, including contradictory points of view and new insights. Analyses of the two independent authors were compared, discussed, and then merged after consensus. Both manifest (evident) and latent (underlying) contents were the primary outcome of analysis to capture a complete meaning of informants. Similar categories were linked to form themes (Figure 1).

In addition, member-checking sessions were conducted with the informants at the interpretation level (during category formation) of the analysis to make sure the informants’ intended meanings, were well represented in the findings. We excluded some information that was contrary to the informants’ views during member checking sessions. No significant changes were made during these sessions implying that the informants agreed with the emerged themes. We selected appropriate quotes that convey the core theme(s) or essence of a respective category(s). A bilingual person who was an expert in both Kiswahili and English translated the chosen quotes from Kiswahili to English. Back translation according to Brislin [34] was done to ensure that all information was captured and the meaning remains the same in both languages.

### 2.5. Ethical Consideration

Ethical approval was obtained from the Institution Review Board of Muhimbili University of Health and Allied Sciences with Ref.No.DA.282/298/01.C. The potential informants had a good link to the trial site because of participation in the previous phase I/II HIV vaccine trial. Participation in this study was completely voluntary. Informants were well informed about the confidentiality of the information they would provide and that all the audiotapes, transcripts, and written notes would be kept confidentially or destroyed after completion of the study. Informants were informed on their right to withdraw or to continue with the study at any time they wished to. Written informed consent was obtained from all informants before the interview sessions.

## 3. Results

### 3.1. Socio-Demographic Characteristics of Informants 

The mean age of informants was 43.9 years with a standard deviation (±SD) of 6.6. The majority of informants were male (70.8%). Three quarters (75%) were married and more than half (54.2%) had secondary education. More than half (58.3%) had work experience of 10–19 years as police officers (Table 1).

### 3.2. Themes and Categories

Four themes emerged namely: Engaging in risky sexual behavior and practices before HIV vaccine trial, compliance to safer sexual behavior and practices after HIV vaccine trial, post-HIV vaccine trial challenges, and opportunities towards safer sexual practices after HIV vaccine trial. The first theme describes the risky sexual practices and the perception of condom use among the informants before participating in the HIV vaccine trial. The second theme gives information on the possible effects of education sessions on safer sex during HIV vaccine trials and how the education sessions changed their sexual behaviors and practices after completion of the trial. The third theme describes the challenges encountered by informants after the trial and the last theme describes the foreseen opportunities of continuing interventions in promoting safer sexual practices. The themes are supported by nine categories as indicated in Table 2.

### 3.3. Engaging in Risky Sexual Behavior and Practices before the HIV Vaccine Trial

#### 3.3.1. Multiple Sexual Partners

Before participation in HIV vaccine trials, male informants reported that having multiple sexual partners was a common practice due to young age and associated physiological demands. They expressed this demand as a strong sexual desire. They also reported that the person with multiple sexual partners was perceived as having a prestigious personality in their working environment, as many women wanting to have sex with him. To express their manhood, they said they had to have sex with many women regardless of age and marital status. Some of the informants confessed that the strong sexual drive made them not to let a beautiful woman pass without proposing her. This behavior imposed quarrels with their spouses when they were suspected to have extramarital affairs. One informant said he could chase out his wife and bring in another woman if the spouse interferes with his sexual affairs outside the wedlock:

“That moment when my blood was boiling [sexually active], my wife could not tell me anything. You see. I would tell her to get out, another woman would come and stay. I was dangerous. I could not let a woman pass without asking her romantically; I could do anything to get her at any cost” (Male, aged 46, married).

The gender difference was noted in sexual relationships. For women, the number of sexual partners depended on the intentions of the male partner. Most women informants described engaging in the sexual relationship for marriage purposes while men approached women with different intentions. Thus, it was easy for women to cease to the relationship and look for another man who could meet their expectations, though it took a little while to get another man. The searching and re-searching process was not a problem as described by the following informant:

“At that time, we [girls]) were not looking for anything such as money, but genuine love from a man. You see. When the relationship ends, you look for another one. It might take a month or two. Then, you find another one in recreational places such as in the bar or workplaces. You start a new relationship with him and forget about the previous one” (Female, aged 54, divorced).

On the other hand, some men confessed that barmaids, unrated attendants, house girls, and food vendors were the most easily accessible sexual partners. However, they considered these categories of women as a high-risk group of people due to risky working environments. One of the informants who maintained the risky sexual practices narrated:

“Still, my lovers [sexual partners] are barmaid, food vendors, waitresses, people’s house girls [house maids] … house girls and barmaids do not differ significantly because they have multiple sexual partners” (Male, aged 42, married).

#### 3.3.2. Unprotected Sexual Intercourse

Many informants reported having unprotected sexual intercourse with multiple sexual partners. This practice exposed them to the risk of acquiring HIV infection and other sexually transmitted diseases (STDs). They gave various reasons to prove such risky sexual practices. They said some men were involved in unprotected sex in an unexpected environment, particularly when it comes to casual sexual partners. They said the nature of the work environment also could hinder access to protective gear such as condoms. This contributed to unsafe sexual practices as narrated below:

“You know, sometimes it [sexual intercourse] can happen as an accident. As I told you how our bodies are created; you find yourself in a situation if you do not practice it [sexual intercourse], you may no longer meet the same sexual partner again. Looking for access to that protective gear [condom], gaining access to such places is very difficult. Otherwise, the act of searching for a condom can undo the whole process of practicing sex”(Male, aged 39, married).

Informants expressed a low understanding of the risk of acquiring sexually transmitted infections, as they believed a smart looking person cannot have any infectious disease. Thus, they engaged in unprotected sex as they trusted the partners by physical appearance as evidenced below:

“It means, once you get her, you believe by looking at her by naked eyes, that this person… cannot have those [HIV and AIDS], cannot have any infection. See how she is! So lovely! So you believe by looking at her, you look at her carefully then you believe that she does not have the infection!” (Male, aged 52, married).

#### 3.3.3. Perceived Barriers to the Use of Condoms

Despite the high risk for infection due to multiple sexual partners and unprotected sex, most informants expressed to be knowledgeable about measures on HIV prevention specifically the use of condoms. However, accessibility and perceptions of condoms were reported to affect their effective use as described in the following sections:

- Inability to access condoms

Some informants argued that the availability of condoms should be made as a service and not as a business particularly in the rural areas. They compared the income of people in rural and urban areas and noted a difference. They reported that peoples’ ability to buy condoms in urban areas is high because they can pay the cost of the guesthouse for sexual intercourse. However, they noted a difference in the rural areas where sexual intercourse was often taking place in the bushes (clusters of shrub appearing as trees) as expressed below:

“In the city, I believe there is enough money to go to the guest house. In the village, most of the sexual intercourses are unprotected sex and take place in the bush and the forest. It is very rare for a married person to walk with a condom. “Will you be able to think of a condom when you find a woman in the bush?” (Male, aged 42, Married).

Informants also commented that the cost of condoms depends on the type of condoms in the market. They reported that some condoms are very expensive while others are cheap. From the informants’ perspective, these price differences was a barrier in the accessibility of condoms though the quality was said to be the same in both types of condoms as expressed:

“These Salama [condoms] seem to be very cheap. Now, these are the things that people are going to pursue more than the other types of condoms. LifeGuard condom is so expensive. It is sold at TZS 3000 (1.30 USD), TZS 5000 (2.16 USD), up to TZS 10,000 (4.32 USD), according to brand names. Now as for the average person, who does not understand, he may stop using condoms, though, in my opinion, all condoms have the same quality” (Male, aged 36, married).

- Perception of condoms

Many informants described not using condoms for different reasons. The most cited reason was that condom decreases sexual pleasure during the sexual act. They reported that condoms prevent skin to skin sensational feeling during sex. They emphasized that during sexual intercourse, skin-to-skin contact produces mutual warmth desired for maximum stimulation to both partners:

“I used condoms in the past. Using a condom during sexual intercourse is different from having sex without a condom because the other person does not feel the warmth. So when you do not use a condom, the taste of sexual contact and the warmth of a woman and a man are present but not the same as if you are using a condom” (Male, aged 59, married).

Women informants also accepted the dissatisfaction that using condoms does not give the desired sexual pleasure due to a lack of comfort during sexual practices. They emphasized the expected sexual pleasure is lost when using a condom. One informant expressed this:

“You know that in making love you expect to find comfort and happiness. Now if you use condoms, then I feel not getting the thing I am supposed to get” (Female, aged 39, separated).

Most male informants expressed a lack of confidence and power to talk about using protective gear during sexual contact. They felt shy towards women when dating. Therefore, the decision to use protective gear remained under the discretion of a female partner. It was also reported that telling the partners to use protective gear could mean the partner is a prostitute and therefore decrease the desire for practicing the planned sexual intercourse. One of the participants revealed this:

“In the past, telling the woman to use a condom was a little difficult. Also, when he [proposer] gets her, he can’t have a decision. The decision is with the woman. She may say: ‘do you see me as a prostitute? So you wear a condom with me?’ You find he [proposal] lacks the confidence and leaves the condom aside, and continues practicing unprotected sex” (Male, aged 49, married).

### 3.4. Compliance to Safer Sexual Behavior and Practices Post-HIV Vaccine Trial

Many informants expressed a change in sexual behavior after participation in the HIV vaccine trial. This change in sexual behavior was the result of the education sessions received during the HIV vaccine trial. They reported changes in the form of strengthened marital relationships, increased understanding of safer sex practice, and regular HIV testing with extramarital partners as detailed in the following sections.

#### 3.4.1. Strengthened Marital Relationship

Many male informants expressed increased time for staying with the family at home. After joining the HIV vaccine trial and having been educated about safer sex practice, they realized no difference between sexual pleasure obtained from marital and extramarital sexual intercourse and hence decided to stick to their spouses. Some behavioral changes such as abstinence from alcohol were also described to strengthen sexual relationship ties as evidenced below:

“This project [HIVIS 03 HIV Vaccine trial] taught us many things. It facilitated the understanding of marital relationships and the avoidance of engaging in relationships with women outside the marriage lock. I have also forgone alcohol stuff, which was associated with risky sexual practices, and remains only with my wife. This is because I do not see what I am looking for outside marriage… it is the same design [sexual relationship] that I have here at home” (Male, aged 43, married).

Other informants reported that they decreased episodes of arguments related to infidelities. They said that women were curious about the sexual behavior of their male partners such as coming home late and other issues related to lack of faithfulness in marriages causing frequent quarrels and disharmony among the spouses. After the HIV vaccine project, many men changed the habits and focused on the marriage relationship as accounted by the following male informant:

“I had frequent arguments with my wife about marital infidelities before joining the HIV vaccine trial. I feel that was something a bit foolish. Since then, I have been close and in a happy mood with my wife all the time” (Male, aged 42, married).

Many informants testified to have time to sit with their wives and children exchanging ideas. They reported this harmony had never happened before joining the HIV vaccine trials. Before the vaccine trial, they used to eat somewhere else and get home late. Also, they did not consider even the welfare of children including school work. These things changed after getting knowledge about sexual matters during the vaccine trial seminars as evidenced below:

“After attending several education sessions on safer sexual practice during the HIV vaccine trial, I found that engaging in relationships outside marriage is just a loss. Also, my children were missing many things from me. It is now funny for children to stay close to their dad [me], and review their school activities, you see” (Male, aged 46, married).

#### 3.4.2. Increased Understanding of the Safer Sexual Practice

Many informants expressed an increased understanding of safer sex practice after the vaccine trial. The sensitization meetings before HIV vaccine trials helped them to evaluate their sexual behavior and feel that it was a risk for them to acquire HIV and other STIs. Some participants described that self-awareness came after they tested for HIV status as part of the criteria to participate in the study. One informant who had multiple sexual partners before joining the trial expressed:

“After participating in sensitization workshops, I decided to give up the habit of having extramarital sexual relationships. Nevertheless, there were no fundamental reasons that led me to have sex outside of marriage. I enrolled in this research, got a lot of training sessions, and decided to stay on this correct path” [stable marital relationship] (Male, aged 42, married).

Some informants described that sexual issues were no longer consuming much of their time. They voiced that the education received had added knowledge that was not apprehended before. Again, they learned that life was difficult and could not afford to have extramarital affairs; rather, they would invest much time in protecting and maintain their health as depicted from the following quote:

“I have significantly reduced my sexual impulses toward other women. Now, it does not take much of my time anymore because, by nature of life, it is difficult to have multiple sexual relationships” (Male, aged 36, married).

As sensitization workshops and education emphasized safer sexual practice, informants expressed a greater understanding of side effects caused by practicing unprotected sex. The sessions given by experts provided precious opportunities to learn and understand many things related to their health. They further noted that for someone who had not received education about sex and HIV transmission, would be doing things differently. The use of illustrations and videos was reported to be educative and helped them to remember well what was taught. They emphasized that educators also had effective teaching skills that facilitated learning among participants. One of the informants expressed this evidence:

“For someone who did not understand about sex and HIV transmission, he/she cannot understand anything. So he will be doing something different. However, if he gets the education like we got, with video illustration from Dr. X [The Principal Investigator], then he can easily understand. So when you look at those disease side effects [Sexually transmitted diseases], even the intention goes back” [Hesitate to have unprotected sex] (Male, aged 43, widowed).

Many informants reported having gained enough knowledge about safer sex practices such that they could teach others how to practice safer sex. They said they could teach not only the spouse but also the young people in the community. They emphasized that, because participants got an opportunity to attend and understand what was taught in the sensitization sessions, it was important to educate others who had not received such valuable education as stated below:

“It helped me to understand and educate the community. There was a time when we were educating the police community about safer sex such as the use of condoms, being faithful to the spouse to avoid sexually transmitted infections. The project [HIVS 03] increased our understanding of the safer sexual practice to educate our families, even our children” (Female, aged 54, divorced).

#### 3.4.3. Regular HIV Testing with Extramarital Relationships

Another fundamental behavioral change reported after the trial was the increase in the tendency to test for HIV status with the sexual partners outside of wedlock. Even though there were different perceptions in the use of condoms among the informants before the HIV vaccine trial, there was an increase in awareness of HIV testing with extramarital partners after the trials. They said that sometimes they tended to go for testing to ensure that they know their health status before and during extramarital sexual relationships as evidenced below.

“We do regular HIV testing because we do not use a barrier such as condoms or whatsoever. But once in a while, we tend to go together [with extramarital sexual partners] testing for HIV, making sure our health status is okay” (Male, aged 42, married).

Other male informants commented that, whenever they wanted to establish a new relationship outside the marital realm, they had to sit down, talk, and agree with the new partners that they should undergo HIV testing. They said if the female partners show hesitancies, it was the sign that the partner does not care about her health status. They emphasized that accepting HIV testing for the partners was a predictor of continuing with the relationship or ending it as in the statement below:

“When initiating a new relationship, I always talk to her [potential sexual partner] for possible HIV testing, telling her, how do you feel about doing this? [Testing for HIV]. I listen to her opinion first. It makes me decide whether to be with her or not. Of course, there is someone you can tell, surprised, oh! no; if she starts expressing that way, then why do you keep up with someone like her? Someone seems not to care” (Male, aged 36, married).

Other informants insisted that having sex outside wedlock was unavoidable, and thus performing HIV testing was common. They reported that when they had to go out of their marriage they had to have blood tests, to check their health status. One male informant described that he had to walk with the HIV test strips [SD-Bioline] obtained from his friend who was a health care worker. He pleaded to test for HIV with casual sexual partners. This was important to ensure that he would not become infected with HIV:

“I have to satisfy myself from tests [HIV tests], eh ... That I know my partner, the status of her health, and she also knows my health status, eh ... After you [I] have satisfied yourself, twice three times, then I can indulge in those relationships. But without that, I don’t need to [engage in sex], eh!” (Male, aged 52, married).

### 3.5. Challenges after the HIV Vaccine Trial

Informants experienced HIV vaccine-induced seropositivity and HIV related stigma as the main challenges during and after participation in the vaccine trial. In addition, some perceived HIV protection from participation in the vaccine trial that changed their sexual behavior as detailed in the following sections:

#### 3.5.1. Experienced HIV Vaccine-Induced Seropositivity

Informants described problems related to reactive HIV test results. They said when they tested positive for HIV, the results imposed disharmony between the couples. They said since they were already instructed about possible vaccine-induced seropositivity, they were aware of not to attempt testing for HIV in health facilities other than the HIV vaccine trial unit. They reasoned that not all facilities were equipped with the recommended confirmatory HIV tests. In some incidences, some of the volunteers forgot about such precautions and got tested in peripheral facilities as evidenced by the following informant:

“There was a time, I went for an HIV test with my wife in a peripheral health facility, they found me HIV positive. When I told the doctor about the vaccine trial I participate in, he said, ah! Dr. X’s [The Principal Investigators] study, let me write you another test. Then that other test [PCR Test], showed that I was HIV negative. Even the wife’s blood pressure returned to normal [spouse’s worries lessened]” (Male, aged 42, married).

Many informants reported that they did not usually go for an HIV test if they got casual sexual partners or when instructed by the regular extramarital partners to do so in fear of testing HIV positive. They argued that it was difficult to convince the sexual partner that they are HIV negative while the test result would show seropositivity. They said, in general, there was no need to have HIV testing unless there was a clear explanation that could satisfy the partner. This created tension and dilemma among partners during sexual practice as stated in the following example:

“I have an extramarital partner, with whom I once had sex. But now she always bothers me to check for HIV status. I always tell her that when I get the time, I will come and test. But I just hang around without going, because I see no specific reason for testing. Suppose I go to test for HIV, the test they call a simple test. When I test positive, what will she think of me?” (Male, aged 42, married).

Other informants narrated that there was an occasion when they went for an HIV test with their spouses. Unfortunately, one of the spouses tested HIV positive, that time he forgot that he would test positive in the ordinary health facilities. They went to another health facility and found the same test results. This situation caused marital conflict. The wife believed her husband was hiding his actual HIV status. The man decided to go to the HIV vaccine trial unit to ask the project team to rescue his marriage because they were in a big conflict as narrated below:

“I told them [project staff], my marriage is breaking up there. I decided to bring my wife here [HIV vaccine trial unit], and we were checked for HIV together. Then she was satisfied after I tested HIV negative…But before that, I lived with this challenge for the first 2 years trying to go to different places to test for HIV (Male, aged 46, married)”.

The informants were also instructed that during the HIV trial seminars they should not donate blood for transfusion. They reported it was difficult to convince the people around who traditionally knew that close relatives are the first to donate blood for transfusion to their patients. This was also mentioned as a challenge to one of the participants who had a sick child as expressed below:

“My son had anemia, you see. We went to the hospital for treatment. The doctor told me that I should donate blood. But that moment, the research team instructed me not to donate blood, okay. I was in a big dilemma. Another problem was when the people began to tell me, ‘As a parent, why don’t you give blood to your son?’ One does not directly tell you that you have been infected with HIV “(Male, aged 46, married).

#### 3.5.2. Perceived HIV Protection after Vaccine Trial

Informants reported that they suspected some people might have joined the HIV vaccine study with different expectations. They said after vaccine trial and unblinding, some volunteers turned around and started having multiple sexual partners. They asserted that at first, they protected themselves but after discovering that they had been vaccinated with the vaccine, they turned to a self-confident group as evidenced by one of the informants below:

“I believe the vaccine protected me. This is because it happened to me; I remember there was a lady who was a police officer. The lady was said to be infected with HIV. This happened one day at a party, I had sex with her without a condom. When I arrived at the office, the next day people were saying that the girl was infected with HIV. I was worried and went for an HIV test … and I was negative [unreactive to HIV test]. So I believe the vaccination helped me” (Male, aged 36, married).

However, an informant who believed that the vaccine was under investigation criticized one of the vaccine trial volunteers who used to cheat women. He was persuading them that, because he was a volunteer in the HIV vaccine project, he had immunity against the HIV infection as he received vaccination during the project. The volunteer who witnessed the scene censured angrily:

“I turned and looked at him, and told him to stop deceiving them [women]. I told him ‘you just fool yourself and your stupidity. Do not deceive innocent people, you have not received a vaccine but that was a trial’. The man looked at me furiously because I told him the truth” (Female, aged 48, widowed).

Some informants expressed uncertainty about the vaccine they received during the trials. Nevertheless, later, they realized the challenge they were facing was a matter of confidence that they had received the vaccine. Informants had tested for HIV status several times during the trial, but naturally, some trial volunteers believed that the vaccine might be working that is why they tested negative. This perception faded out in some of the volunteers towards the end of the trial as stated below:

“At the end of the day, they realized it was not a preventive HIV vaccine that caused negative HIV tests because it was revealed that some were given a placebo” (Male, aged 42, married).

Other informants were wondering why they were resistant to HIV infection even though they practiced unsafe sex with suspected HIV infected partners. They suspected that either the vaccine they received or their specific blood group might have enhanced the immunity of their body. They proclaimed that unlike other blood groups, blood group O positive provided immunity against many infections including HIV. One of the informants asserted the following statement with uncertainty:

“Now I think there are two things. Either the vaccine helped me or my blood group. I usually hear my blood group [O positive], is immune to infection than other blood groups. Therefore, these two things give me confidence that I am protected. However, between the two, I have no idea which one is helping me” (Male, aged 43, widowed).

### 3.6. Opportunities towards Safer Sexual Behavior and Practices

#### 3.6.1. Continuing Safer Sex Educational Interventions

Informants emphasized that they need to meet frequently to be reminded about the safer sexual practices and a follow up on how the volunteers are behaving after participating in the vaccine trial. However, they cautioned that the trial participants were many and they are scattered in different places, and thus it may be difficult to know the whereabouts. They added that frequent education could also help in educating others on how to practice safer sex and to know their health status. One participant said:

“I think regular seminars will help because trial volunteers are scattered in different places. Seminars can bring us together to learn from each other and check our health status and sexually transmitted diseases as well. Regular seminars will reduce those negative thoughts” (Male, aged 39, separated).

Another informant emphasized to have access to continued education despite the level of knowledge they had during the previous vaccine trials:

“Education must continue so that one does not just sit dormant and forget. That is, education, education. This is because everyone knows the use of a condom. How is a condom being used, why should I use a condom? I use condoms to avoid A, B, C. Still reminding them is important. You see!” (Male, aged 39, married).

Many informants said they would advise the HIV vaccine research team to continue educating the previous HIV vaccine cohort specifically those who had increased the number of sexual partners due to the perception that the vaccine could have protected them from HIV infection. They emphasized and compared the education with an armed soldier that any time is prepared for attacks from the enemies. Thus, they insisted that frequent reminders would ultimately reshape the difficult behavior against safer sex. One informant stated:

“It is just to continue providing them with education on how to have safe sex, not feel confident that the vaccine is in the body, that they cannot get the infection. Be that as long as we, soldiers say, when you take up arms, when you hold a gun, even if it does not shoot in, you should know it has a bullet” (Female, aged 54, divorced).

#### 3.6.2. Involvement of the Community in Promoting Safer Sexual Practices

Several informants described the importance of increasing awareness of safer sex practices among potential participants of HIV vaccine trials. They said that people take a longer time to change their sexual behavior as compared to the trial period. They suggested that one of the methods to reach people could be passing through public places including government and private institutions premises such as offices, schools, and other public fora with advertisements:

“I am just saying that, first, the experts, devote themselves, either to talk at public gatherings or sensitize and give information in the institutions. For example, in offices, at school, even in rural areas, and in public forums. With the program, we look for leaflets, fliers that educate the young adults about sexuality, and avoid disease” (Male, aged 47, married).

Other informants reported that sexual education was indeed widespread, but it had not reached many beneficiaries. They said sexual education and sexuality among young adults and adolescent is given through different media. However, many people do not get it for various reasons including being reluctant to follow information related to health and sexuality in some media. Thus, they suggested continued support among this group, as they might be potential HIV vaccine trial volunteers as described below:

“You know the problem is that Tanzanians are hesitant to listen to the media, but they enjoy listening to music. Nevertheless, when you talk about HIV/AIDS, someone will turn on the radio searching for another station. On the other hand, if you put up a movie for him on TV, he will sit and watch. But for basic things like HIV/AIDS, someone will not be interested. Therefore, the community’s understanding of these issues is still low” (Male, aged 42, married).

Overall, the informants emphasized that education about safer sex should continue and health professionals should not give up on the matter. They verbalized that safer sex education should be like a national anthem. Just like a president, in every great event when addressing the nation should sing the song as stated by an informant below:

“In my opinion, education should continue and let us not give up, and be like a national anthem. As you see the president walking into a place, the song is vocal. A specific slogan should be made that people, a priest, an ordinary man, or a leader of a country may use. Speaking even if it is in church after finishing they should talk about sexual issues in general, and testing for HIV” (Male, aged 36, married).

Other informants insisted on the continuity of imparting information related to sexuality among young adults. They added that education about safer sex and HIV should be cautiously given in a positive and non-threatening way particularly for those who are HIV positive taking antiretroviral therapies (ARTs). One of the informants paraphrased this in the following statements:

“Education about safer sex and HIV should be instilled in a positive way among youths. This is because there are people taking medicine [ARVs]. When you start giving HIV negativities, will affect him psychologically. That is, if it is HIV or safe sex education, it should all be discussed positively so that there is no threat” (Female, aged 37, married).

In general, the informants describe the foreseen opportunities for continuing interventions in promoting safer sexual practices. The use of various education platforms such as flyers and pamphlets can enhance understanding of safer sexual practices among young adults in the community.

## 4. Discussion

This study analyzes the sexual behaviors and practices before and after a phase I/II HIV vaccine trial among informants who took part in the respective trial. Specifically, the study reports high-risk sexual behaviors and practices including unprotected multiple sexual relationships before participation in the HIV vaccine trial. After participation in the HIV vaccine trial, informants reported strengthened marital relationships, increased understanding of safer sexual practices, and frequent HIV testing with sexual partners as the positive changes. However, the consequences of vaccine-induced seropositivity were the complaints that adversely affected the sexual relationship among trial volunteers. Additionally, the perceived HIV protection among volunteers was another challenge reported in this study. Continued education intervention and community engagement in the provision of safer sex education were suggested as opportunities to enhance safer sexual practices in future trials.

### 4.1. Engaging in Risky Sexual Behavior and Practices before the HIV Vaccine Trial

The fact that informants practiced risky sexual behaviors before participation in an HIV vaccine trial implies that most of them were unaware of safer sexual practices. This may be described by individual risky sexual behavior. Individual risky sexual behavior can be attributed to the young age of the trial volunteers and the negative perception of HIV protective measures specifically the use of condoms. During the phase I/II vaccine trial, the volunteers were aged between 18 and 40 years, indicating they were at higher sexual desire compared to other age groups as reported in another study [35]. In the context of our study, it is obvious that males appear to have higher sexual impulses compared to females. The reason for this high sexual impulse is different between males and females [36] and may be attributed to physiological factors. Despite risky sexual practices due to their young age as reported by the informants in the current study, the behavior could be changed if appropriate safer sex education is provided as reported in the previous studies [28,29]. Unprotected sexual practices among informants could have been caused by the attitudes and perceptions towards condoms use [37,38]. Many studies have advocated the use of condoms with multiple sexual partners as an effective method of prevention of HIV infection [3,39,40,41]. However, our findings revealed that the inaccessibility of and perceptions towards condoms use were the main obstacles in practicing safer sex. Despite the low price/cost of condoms in Tanzania, our informants did not use condoms with new sexual partners of unknown HIV status. This indicates that availability is not the single barrier for accessing and using condoms. Mbubyazi et al. reported that short supply of condoms, shyness to be watched while purchasing condoms and prevailing social perception of condoms to have low/no protective efficacy are the reason for unprotected sex among the risk population in Tanzania [42]. Lack of confidence to negotiate condom use and the perception that using a condom is the sign of prostitution are also reasons for unprotected sex in our study. A review of the literature reported a similar perception of aversion to condom use due to a lack of confidence [43]. For the preparation of the HIV vaccine cohort, effective safer sex education is required to remove the perception and attitudes towards condom use. The perception that condoms decrease sexual pleasure can be attributed to psychological experience and the strength of partner relationships. This condom experience and perception of decreased sexual pleasure have been reported in other studies [44,45,46]. To counteract this effect, Hensel et al. asserted that sexual pleasure can be increased in conjunction with specific relational, physiological, and condom perceptions [47]. However, more studies in this area are required to explore the relationship context when assessing condom use experience and sexual pleasure.

### 4.2. Compliance to Safer Sexual Behavior and Practices after the HIV Vaccine Trial

The reported changes in sexual behavior and practice because of participation in the HIV vaccine trial implies that the HIV vaccine trial interventions were useful. This might be due to the effectiveness of the educational seminars and training on safer sex practice which brought about the changes in sexual behavior [28]. The reported improved communication among spouses is one of the factors that strengthen marital relationships in our study. This might be attributed to individual maturity and understanding of safer sexual relationships’ matters through trial interventions. Swensen and colleagues reported similar findings in the US that older married couples expressed fewer marriage problems than younger couples [48]. Contrary to our findings, Velten and Margraf in Germany found that neither age nor relationship duration may be the significant predictors of marital satisfaction and stability [49]. The difference may be attributed to socio-cultural differences between Tanzania and Germany. The high sexual desire that leads to extramarital relationships disrupts family relationships causing frequent conflicts among the couples. As reported, coming home late appears to be a source of conflict among couples. Safer sex education provided during the vaccine trial might have improved the awareness of these factors and increased trust among the spouses that contributed to stable marital relationships. However, other factors about marriage stability and satisfactions need further investigation.

Concerning the increased understanding of safer sex among the informants implies that informants had limited knowledge about safer sex practice before participating in the trial. The series of educational sessions and workshops during the trial might have increased their knowledge of preventive measures against HIV and other STIs. Previous studies in Dar es Salaam, Tanzania reported similar changes after the interventions [13,28]. The use of visual aids to demonstrate the consequences of unprotected sex during the vaccine trials may have promoted a deeper understanding of the learning contents. A systematic review shows that educational intervention focusing on risk reduction is an effective tool with a long-term impact on behavioral changes among the high-risk sexual groups [50]. Our study emphasizes the significance of comprehensive information seminars and education before potential volunteers embark on the vaccine trial. Additionally, the invitation of the spouse in the workshop and seminars has greater impacts on mutual understanding of the topic even after the session.

The increased frequency of HIV testing with extramarital sexual relationships can be described by an increase in confidence after conducting a series of HIV tests during the vaccine trial. Before the trial, the study participants rarely tested for HIV status. After the experience of repeated HIV tests during the vaccine trial, the frequency of testing was fueled by distrust from the sexual partners, the need to know individual HIV status, availability of HIV testing equipment, and the training seminars that were provided during the trial. Similar motivators have been reported in other countries [51,52,53]. A systematic review revealed that perceived testing benefits, knowledge of testing sites, and knowing someone with HIV is the motivator for HIV testing [54]. The present study informs that counseling and behavioral risk assessments during the follow-up visit helps to increase awareness of personal risk-taking behavior.

### 4.3. Challenges after the HIV Vaccine Trial

The fact that vaccine-induced seropositivity (VISP) emerged as a challenge among the trial volunteers implies that inadequate information was provided about the effects of VISP that could be detected after the HIV vaccine trial. In this study, non-vaccine trial participants misinterpret VISP as a sign of HIV infection that affected sexual relationships. Testing HIV positive due to vaccine trials is a distressing phenomenon that causes conflicts in both married couples and extramarital sexual relationships [55]. Commonly, participants are informed about VISP before participation in the vaccine trial. However, the long term follow-up of participants to identify social impacts VISP including sexual relationships has not been implemented in many studies [56,57]. A joint effort is needed to addresses these challenges, particularly after the vaccine trial. A review of the literature shows that VISP can be addressed by the collaborative approach among the trial participants, researchers, and healthcare providers [26]. The finding in this study implies that future HIV vaccine trials should have priority strategies for providing the needed social support to volunteers after the trial to reduce the effects of VISP such as disrupted sexual relationships.

Although this study reports changes in behavior towards safer sexual practice, some informants believed to be protected from HIV infection after the vaccine trials. Testing negative for HIV despite the number of incidences of unprotected sex might have contributed to this perception among the informants. In addition, the low understanding of how vaccine trials works make them believe that the experimental vaccine could have prevented them from HIV infection. This is in line with the systematic reviews that showed an increase in risky sexual behavior after HIV preventive trials [14,58]. Contrary to this, other studies have reported a greater understanding of vaccine trials with a reduction in sexual behaviors [20,59]. On the other hand, the perception that individual blood group O positive protect the trial participants from HIV infection lacks scientific evidence. Anecdotal evidence has shown that blood cell antigens and rhesus factor C have influence and interaction with HIV [60,61,62]. This is a novel and interesting area to be investigated in the future.

### 4.4. Opportunities towards Safer Sexual Behavior and Practices

Our study revealed a need for continued safer education and intervention among the trial participants. This suggests that educational interventions will not only help to remind the trial participants what they learned during the trial but also help to understand their persistence risky sexual behavior. It is also important to note that follow up of study participants is crucial for the assessment of any health problems related to HIV vaccine trials and provides appropriate interventions [19,28,63]. Even though the informants were appropriately prepared for phase I/II HIV vaccine trials according to good participatory practice [64], they still need more information regarding the safer sex practice.

The suggestion that the community should be fully engaged in safer sex education indicates that change in behavior is a process that needs effective community participation. Different platforms including capacity building, use of community fliers, and other social gatherings can facilitate sexual and behavioral changes. A study in the same cohort reported a similar suggestion that wider community dissemination of information and post-trial feedback is essential to alleviate concerns among the participating communities [65]. Continued active community involvement in HIV vaccine trials is an important strategy proposed in our study to improve and promote sexual and behavioral changes. It is reported that community engagement and HIV vaccine trial participation are inseparable [66] but the vaccine information should be cautiously given to avoid confusion among the target population. The use of various media of instruction such as videos and pamphlets can enhance understanding of safer sex practice among young adults in the community.

This study has the following limitations: Some informants might not have expressed their actual sexual practice due to gender differences in the disclosure of sensitive topic content [67]. Women may have under-reported sexual behavior and male over-reported number of sexual partners to express their masculinity. However, the author tried as much as possible to pose several probing questions making the informants free to express their experience of sexual practice. In addition, we selected informants based on the availability in Dar es Salaam. Some potential informants who had moved or transferred to other working stations outside of Dar es Salaam regions could have given different views and experiences on sexual behaviors, thus influencing the current findings. However, the information obtained is still valid for transferring to similar settings.

## 5. Conclusions

Overall, this study revealed important changes in participants’ sexual behaviors and practice after phase I/II HIV vaccine trial. Low understanding of HIV and other STIs and misperception of condom use contribute to the multiple relationships and unprotected sexual practices among study informants before participation in the HIV vaccine trial. The series of educational seminars during the HIV vaccine trial improved the understanding of safer sex practice among the trial volunteers leading to strengthened marital relationships and frequent HIV testing. However, vaccine-induced seropositivity and the false perception that the experimental vaccine was protective against HIV transmission are important challenges that need appropriate intervention. Future HIV vaccine trials need to focus on education to the communities where the vaccine trial volunteers come from to enhance understanding of the safer sexual practice.

## Figures and Tables

**Table 1:** Socio-demographic characteristics.

Socio-Demographic Characteristics	Number (%)
Age: mean = 43.9 years, (SD = 6.6) at the time of this study	
Gender	
Male	17(70.8)
Female	7(29.2)
Marital status	
Single	1(4.2)
Married	18(75.0)
Divorced	1(4.2)
Separated	2(8.3)
Widowed	2(8.3)
Level of education	
Primary	11(45.8)
Secondary	13(54.2)
Work Experience (Years)	
10–19	14(58.3)
20–29	7(29.2)
30–39	2(8.3)
≥40	1(4.2)

**Table 2:** Themes and categories.

Themes	Category
Engaging in risky sexual behavior and practices before the HIV vaccine trial	Multiple sexual partners (11/24)
	Unprotected sexual intercourse (14/24)
	Perceived barriers to the use of condoms (17/24)
Compliance to safer sexual behavior and practices after HIV vaccine trial	Strengthened marital relationship (8/24)
	Increased understanding of safer sex practice (13/24)
	Regular HIV testing with extramarital partners (17/24)
Challenges after HIV vaccine trial	Experienced HIV vaccine-induced seropositivity (6/24)
	Perceived protection after HIV vaccine trial (9/24)
Opportunities towards safer sexual practices after HIV vaccine trial	Continuing safer sex educational intervention (15/24)
	Involvement of the community in promoting safer sexual practices (10/24)
